# Maintenance of efficacy of canakinumab in SJIA at the individual patient level in a 12-week pooled dataset

**DOI:** 10.1186/1546-0096-12-S1-P69

**Published:** 2014-09-17

**Authors:** A Ravelli, HI Brunner, N Ruperto, P Quartier, A Consolaro, N Wulffraat, K Lheritier, C Gaillez, A Martini, DJ Lovell

**Affiliations:** 1 Instituto Gaslini-PRINTO, Genova, Italy; 2 PRCSG, Cincinnati, USA; 3 Necker-Enfant Malades Hospital, Paris, France; 4 UMC Utrecht, Utrecht, Netherlands; 5 Novartis Pharma AG, Basel, Switzerland

## Introduction

Recent advances in the management of SJIA considered the induction or maintenance of inactive disease according to the JADAS 10-CRP (J10) or 27-CRP (J27) scoring system [1,2]. The maintenance of efficacy of canakinumab (CAN), a selective, human, anti-IL-1β monoclonal antibody, was demonstrated in the withdrawal phase of 2 phase III trials [3], but was not evaluated at the individual level.

## Objectives

To evaluate the maintenance of efficacy at the level of the individual patient from Week 2 to 12, using the adapted ACR-JIA response criteria (aACR) as well as J10 and J27 on the 12-week pooled data set (3 phase III studies).

## Methods

For this post-hoc analysis of the CAN Phase III program in SJIA, the change in disease states between Day(D) 15 and D85 of a total of 178 CAN-naïve patients was assessed. Subjects were 2–19 years of age and had active SJIA at enrollment. This shift analysis considered the aACR response and certain disease activity states as defined using J10 and J27: Inactive Disease (ID), Low Disease Activity (LDA), Moderate Disease Activity (MDA); High Disease Activity (HDA).

## Results

J10 changes during the study period are provided in Table 1. Results for the J27 were very similar to the J10 observations.

**Table 1:** J10 shift analysis table from D15 to D85*

N (%)	Disease State at Day 15*	Disease state at Day 85*			
		
		ID	LDA	MDA	HDA
**ID**	28 (100)	24 (85.7)	1 (3.6)	3 (10.7)	0 (0.0)

**LDA**	20 (100)	10 (50.0)	10 (50.0)	0 (0.0)	0 (0.0)

**MDA**	30 (100)	5 (16.7)	12 (40.0)	10 (33.3)	3 (10.0)

**HDA**	44 (100)	2 (4.5)	6 (13.6)	8 (18.2)	28 (63.6)

*Only patients with both a Day 15 and a Day 85 value are included

The D15-D85 aACR shift analyses, including only patients who had a D15 and a D85 value, likewise indicated that the majority of patients maintained or improved their response: NR (n=32): 12.5% of patients improved; aACR30 (n=14): 0.0% were maintained/78.6% improved; aACR50 (n=21): 33.3% were maintained/42.9% improved; aACR70 (n=36): 25.0% were maintained/58.3% improved; aACR90 (n=26): 30.8% were maintained/57.7% improved; aACR100 (n=34): 82.4% were maintained.

## Conclusion

The great majority of CAN patients either maintained or improved their JADAS status or aACR response level from week 2 to 12. These data confirm the consistent maintenance of efficacy of CAN at the individual level in the first 3 months, irrespective of the measure of response, i.e. aACR criteria or JADAS-derived criteria, and extend previous findings at the study group level.

## Disclosure of interest

A. Ravelli Grant / Research Support from: Pfizer, Consultant for: Abbvie, Bristol Myers Squibb, Novartis, Pfizer, Roche and Johnson & Johnson, Speaker Bureau of: Abbvie, Bristol Myers Squibb, Novartis, Pfizer, Roche and Johnson & Johnson, H. Brunner Consultant for: Novartis, Genentech, Pfizer, UCB, AstraZeneca, Biogen, Boehringer-Ingelheim, Regeneron, Paid Instructor for: Novartis, Speaker Bureau of: Novartis, Genentech, N. Ruperto Grant / Research Support from: To Gaslini Hospital: Abbott, Astrazeneca, BMS, Centocor Research & Development, Eli Lilly and Company, "Francesco Angelini", Glaxo Smith & Kline, Italfarmaco, Novartis, Pfizer Inc., Roche, Sanofi Aventis, Schwarz Biosciences GmbH, Xoma, Wyeth Pharmaceuticals Inc., , Speaker Bureau of: Astrazeneca, Bristol Myers and Squibb, Janssen Biologics B.V.,Roche, Wyeth/Pfizer, P. Quartier Grant / Research Support from: Abbvie, BMS, Chugai-Roche, Novartis, Pfizer, SOBI, Consultant for: Abbvie, Chugai-Roche, Novartis, Pfizer, Servier and SOBI, Speaker Bureau of: Chugai-Roche, MEDIMMUNE, Novartis, Pfizer, A. Consolaro Consultant for: Novartis, N. Wulffraat Grant / Research Support from: Abbvie, Roche, Consultant for: Novartis, Pfizer, Roche, K. Lheritier Shareholder of: Novartis, Employee of: Novartis, C. Gaillez Shareholder of: Novartis, Employee of: Novartis, A. Martini Grant / Research Support from: The Gaslini Hospital, which is the public Hospital where I work as full time employee, has received contributions to support the PRINTO research activities from the following companies: Bristol Myers and Squibb, Centocor Research & Development, GlaxoSmith & Kline,Novartis,Pfizer Inc, Roche, Sanofi Aventis, Schwarz Biosciences GmbH, Speaker Bureau of: Abbott, Bristol MyersSquibb, Astellas, Behringer, Italfarmaco, MedImmune, Novartis, NovoNordisk, Pfizer,Sanofi,Roche, Servier, D. Lovell Grant / Research Support from: National Institutes of Health- NIAMS, Consultant for: Astra-Zeneca, Centocor, Amgen, Bristol Meyers Squibb, Abbott, Pfizer, Regeneron, Roche, Novartis, UBC, Forest Research Institute, Horizon, Johnson & Johnson , Speaker Bureau of: Novartis, Roche.
